# Supplementary material for: Photo-induced semimetallic states realised in electron–hole coupled insulators
Source: Nat Commun. 2018 Oct 17;9:4322. doi: 10.1038/s41467-018-06801-1 (PMC6192982; doi:10.1038/s41467-018-06801-1)
Supplement: Supplementary file 1 — Supplementary Information [file 41467_2018_6801_MOESM1_ESM.pdf]

Supplementary Figures

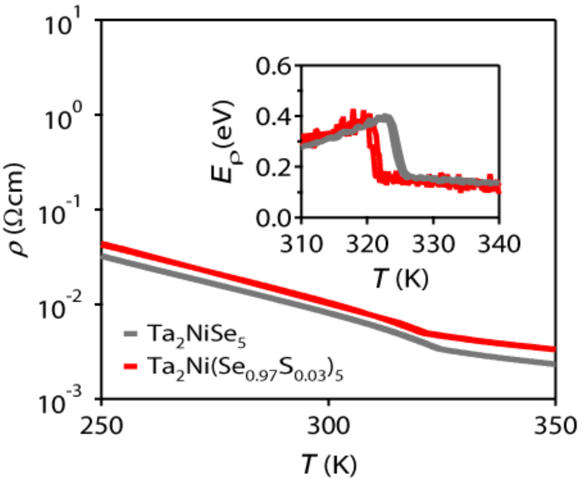

Supplementary Figure 1 | Resistivity data of Ta_2NiSe_5 and $Ta_2Ni(Se_{0.97}S_{0.03})_5$. The results for the pristine and substituted samples are plotted by grey and red lines, respectively. The inset shows the activation energy ($E_\rho = -k_B T^2(\partial \ln\rho/\partial T)$) deduced from the resistivity data. Compared to the pristine sample, the critical temperature of the substituted sample is lower by ~4 K, but the activation energy is almost the same between these two compositions.

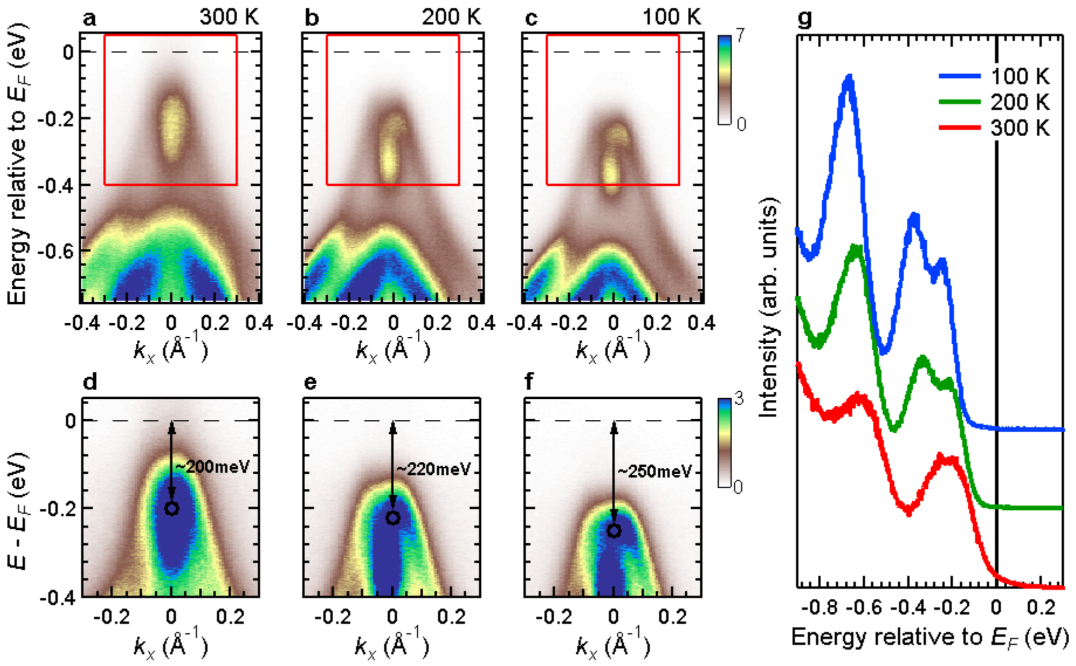

Supplementary Figure 2 | Temperature-dependent ARPES spectra of $Ta_2Ni(Se_{0.97}S_{0.03})_5$. **a–c,** Energy vs. momentum (*E*-*k*) images around the Γ point taken with He Iα resonance line (21.2 eV) at 300, 200, and 100 K, respectively. **d–f,** Magnifications of **a–c** around E_F. **g,** Temperature-dependent EDCs around the Γ point.

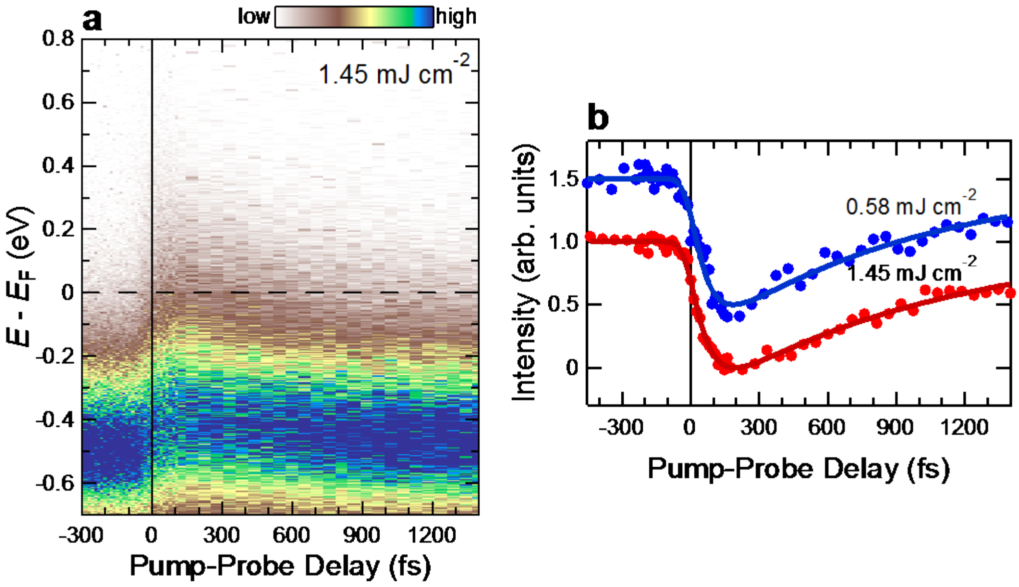

Supplementary Figure 3 | Pump fluence dependence of Ta_2NiS_5. a, TARPES intensity map of Ta_2NiS_5 as a function of pump-probe delay and energy relative to E_F taken with a pump fluence of 1.45 mJ cm^{-2}. **b,** Temporal evolution of the TARPES intensity integrated in [-0.7, -0.5] eV for different pump fluences.

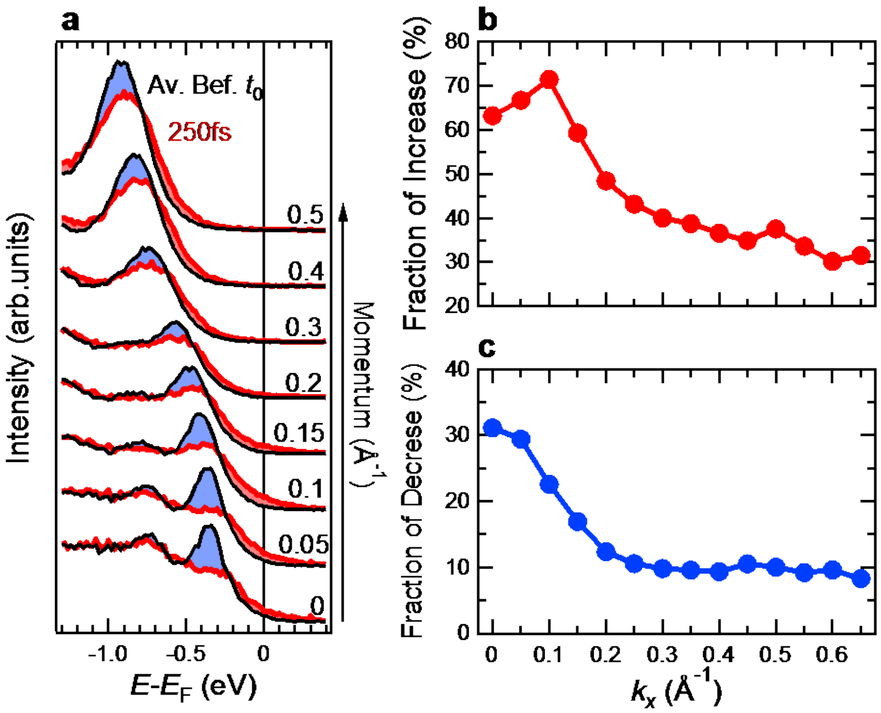

Supplementary Figure 4 | Comparison of EDCs before and after pumping at various momenta. a, EDCs before and after pumping at various momenta. The EDCs before pumping were averaged for several EDCs and the EDCs after pumping were measured 250 fs after pumping. **b, c,** Momentum dependence of the fractions of increase and decrease defined by Supplementary Eqs. 1 and 2, respectively. These plots roughly correspond to the MDC at E_F after pumping and the MDC at the top of the flat band before pumping, respectively.

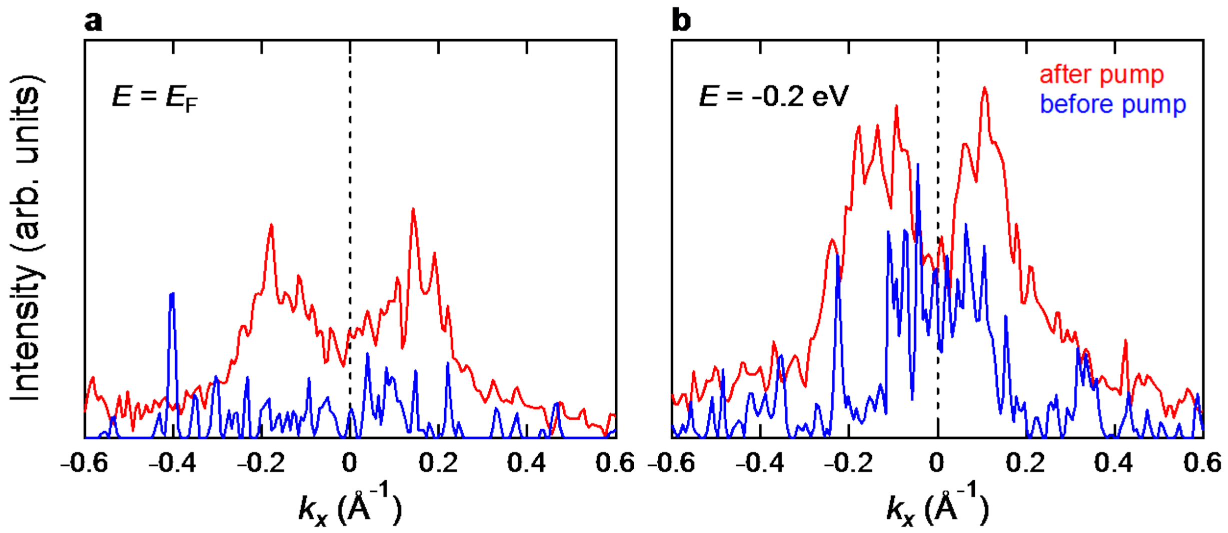

Supplementary Figure 5 | Comparison of MDCs before and after pumping. a, b, MDCs before and after pumping at E_F and E = -0.2 eV (top of the flat band before pumping), respectively, from the TARPES spectra shown in Fig. 4. The energy integration window is ±0.1 eV.

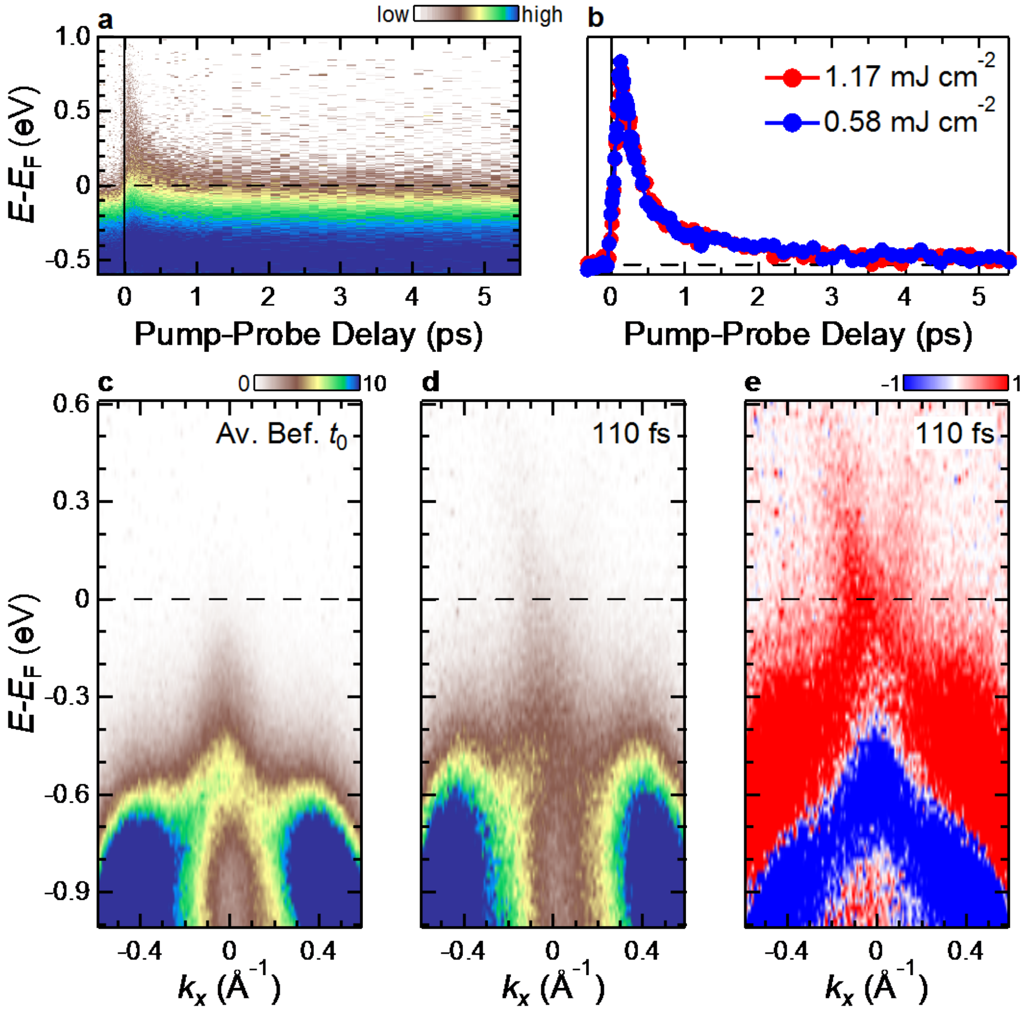

Supplementary Figure 6 | TARPES spectra of Ta_2NiS_5. a, Photoemission intensity map of Ta_2NiS_5 as a function of energy and pump-probe delay. **b,** Temporal evolution of the integrated intensity in the energy interval [0, 1] eV, measured with pump fluences of 1.17 and 0.58 mJ cm^{-2}. **c, d,** TARPES intensity maps as a function of energy and momentum before pumping and at 110 fs after pumping, respectively (see also Supplementary Movie 2). **e,** Difference between the TARPES spectra shown in **c** and **d**. The red and blue regions correspond to the spectra appearing and disappearing after pumping, respectively.

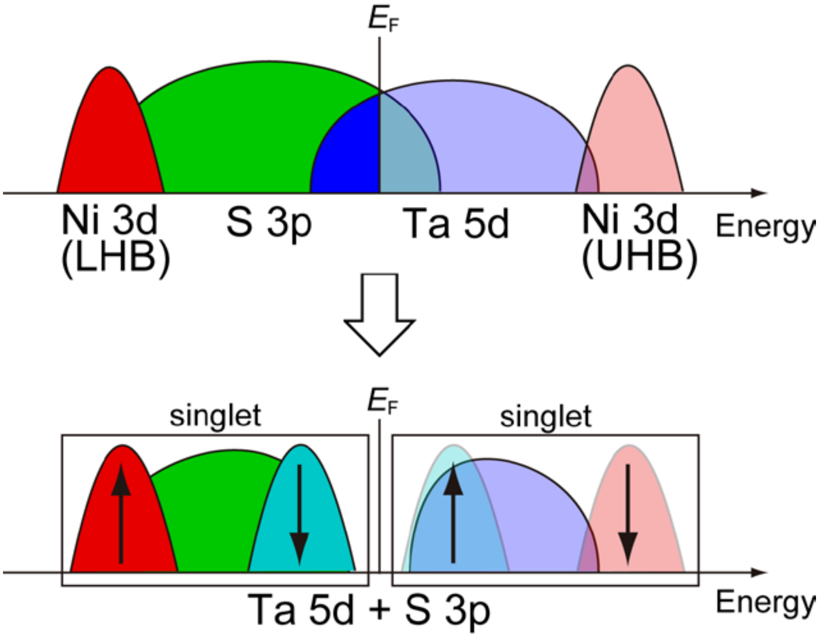

Supplementary Figure 7 | Schematic energy diagram for the possible ground-state electronic configurations of Ta_2NiS_5. Ta 5*d* electrons in the double chains form singlet states with the localized $d^9\underline{L}$ state via hybridization with the S 3*p* orbitals, and the ground state of Ta_2NiS_5 can be viewed as a valence-bond insulator. LHB and UHB denotate “lower Hubbard band” and “upper Hubbard band”, respectively.

Supplementary Note

Definition of fractions of increase and decrease

Supplementary Figure 4 shows the EDCs of $Ta_2Ni(Se_{0.97}S_{0.03})_5$ before and after pumping at various momenta. It is obvious that the spectral weight of the EDC peak corresponding to the flat band before pumping is shifted to the higher energies with increasing momentum. These two EDCs intersect at the lower- and higher-energy sides of the flat-band peak at the points of ω_1 and ω_2, respectively. Using ω_1 and ω_2, the fractions of increase and decrease plotted in Supplementary Fig. 4b and 4c are defined as follows:

$$\text{fraction of increase} = \int_{\omega_2}^{\infty} I(\omega, t = 250\ \text{fs})d\omega \bigg/ \int_{\omega_2}^{\infty} I(\omega, t < 0)d\omega - 1, \quad (1)$$

$$\text{fraction of decrease} = 1 - \int_{\omega_1}^{\omega_2} I(\omega, t = 250\ \text{fs})d\omega / \int_{\omega_1}^{\omega_2} I(\omega, t < 0)d\omega. \quad (2)$$

The fraction of increase has its maximum at 0.1 $Å^{-1}$, which roughly coincides with the location of the Fermi momentum in the photoinduced metallic state. On the other hand, the fraction of decrease has its maximum at the BZ centre, which corresponds to the location of the flat band peak. Therefore, these plots roughly correspond to the MDCs after and before pumping.

Quantitative evaluation of gap quenching time

We used a method similar to that described in Ref. 1 to quantitatively evaluate the number of absorbed photons per Ni atom and the plasma response time after photoexcitation by an 800 nm (1.55 eV) pump pulse.

First, we consider an incident pump fluence of 1 $mJcm^{-2}$ = 4×10^{15} photons per cm^2. It has been reported[2] that the optical conductivity σ and dielectric constant ε_r of Ta_2NiSe_5 at 1.55 eV are ~3000 $\Omega^{-1}cm^{-1}$ and 10, respectively, from which an optical reflectance of 42.8% and an absorption coefficient of 3×10^5 cm^{-1} were obtained. From these values, the absorbed photon density was evaluated to be 7×10^{20} photons cm^{-3}. Considering that Ta_2NiSe_5 has a unit cell volume of 701 $Å^3$ and a density of 4 Ni atoms per unit cell, this is equivalent to 0.12 photons per Ni atom.

In the presence of photoexcited electrons and holes, the plasma frequency ω_p is given by

,

$$\omega_p = \sqrt{\frac{e^2}{\varepsilon_0 \varepsilon_r} \times \left(\frac{n_h}{m_h^*} + \frac{n_e}{m_e^*}\right)} \quad (3)$$

where n_h and n_e are the carrier numbers, m^*_h and m^*_e are the effective masses of the excited holes and electrons, respectively, e is the elemental charge, and ε_0 is the electric constant. The TARPES image of Ta_2NiSe_5 in the photoinduced metallic phase shows that the holes and electrons are fully compensated (n_h = n_e) and the effective

mass of the hole and electron bands is almost equal ($m^*_h = m^*_e = 0.37m_0$, where m_0 is the bare electron mass). Based on these results, the observed quenching time of the flat band $\tau_{Flat} \approx 90$ fs (corresponding to $\omega_p \approx 45$ meV) at a pump fluence of 1 mJ cm^{-2} corresponds to a quantum efficiency (fraction of the number of electron-hole pairs per absorbed photon) of ~1.6%. However, the effective mass of the bands emerging in the photoinduced metallic state might be inappropriate for the evaluation of the number of photoexcited electron-hole pairs. If we use the effective mass of the flat band before photoexcitation (~5 m_0) and assume a quantum efficiency of 21.5%, the observed quenching time of the flat band $\tau_{Flat} \approx 90$ fs at 1 mJ cm^{-2} can be deduced, which is more reasonable.

Supplementary References

1. Hellmann, S. *et al.*, Time-domain classification of charge-density-wave insulators, *Nat. Commun.* **3**, 1069 (2012).
2. Larkin, T. I. *et al.*, Giant exciton Fano resonance in quasi-one-dimensional Ta_2NiSe_5, *Phys. Rev. B* **95**, 195144 (2017).
