# Supplementary material for: Photo-induced semimetallic states realised in electron–hole coupled insulators
Source: Nat Commun. 2018 Oct 17;9:4322. doi: 10.1038/s41467-018-06801-1 (PMC6192982; doi:10.1038/s41467-018-06801-1)
Supplement: Supplementary file 3 — Description of Additional Supplementary Files [file 41467_2018_6801_MOESM3_ESM.pdf]

Description of Additional Supplementary Files

File Name: Supplementary Movie 1
Description: Movie of the temporal evolution of TARPES spectra of Ta_2NiSe_5.

File Name: Supplementary Movie 2
Description: Movie of the temporal evolution of TARPES spectra of Ta_2NiS_5.
